# Attenuation of the Sensing Capabilities of PhoQ in Transition to Obligate Insect–Bacterial Association

**DOI:** 10.1371/journal.pgen.1002349

**Published:** 2011-11-03

**Authors:** Mauricio Henriques Pontes, Kari Lyn Smith, Linda De Vooght, Jan Van Den Abbeele, Colin Dale

**Affiliations:** 1 Department of Biology, University of Utah, Salt Lake City, Utah, United States of America; 2 Department of Biological Sciences, Institute of Tropical Medicine, Antwerp, Belgium; Yale University, United States of America

## Abstract

*Sodalis glossinidius*, a maternally inherited endosymbiont of the tsetse fly, maintains genes encoding homologues of the PhoP-PhoQ two-component regulatory system. This two-component system has been extensively studied in facultative bacterial pathogens and is known to serve as an environmental magnesium sensor and a regulator of key virulence determinants. In the current study, we show that the inactivation of the response regulator, *phoP*, renders *S. glossinidius* sensitive to insect derived cationic antimicrobial peptides (AMPs). The resulting mutant strain displays reduced expression of genes involved in the structural modification of lipid A that facilitates resistance to AMPs. In addition, the inactivation of *phoP* alters the expression of type-III secretion system (TTSS) genes encoded within three distinct chromosomal regions, indicating that PhoP-PhoQ also serves as a master regulator of TTSS gene expression. In the absence of *phoP*, *S. glossinidius* is unable to superinfect either its natural tsetse fly host or a closely related hippoboscid louse fly. Furthermore, we show that the *S. glossinidius* PhoQ sensor kinase has undergone functional adaptations that result in a substantially diminished ability to sense ancestral signals. The loss of PhoQ's sensory capability is predicted to represent a novel adaptation to the static symbiotic lifestyle, allowing *S. glossinidius* to constitutively express genes that facilitate resistance to host derived AMPs.

## Author Summary

Mutualistic insect endosymbionts are known to undergo a process of degenerative evolution that streamlines their gene inventory in accordance with the obligate nature of the host associated lifestyle. Here we show that the mutualistic insect endosymbiont *Sodalis glossinidius* utilizes a two-component regulatory system (PhoP-PhoQ) that evolved to facilitate the regulation of virulence genes in facultative pathogens in accordance with environmental magnesium. While the regulatory targets of PhoP-PhoQ are conserved among *S. glossinidius* and a wide range of bacterial pathogens, the *S. glossinidius* PhoP-PhoQ system is unusual because it has a substantially reduced ability to sense magnesium as a repressing signal. This enables *S. glossinidius* to express genes encoding critical symbiosis determinants within host environments that are rich in both magnesium and antimicrobial peptides. These findings illustrate how a change in the ecology of a host–symbiont association can influence the dynamic functionality of a regulatory system in the symbiont.

## Introduction

Many animals have adopted mutualistic associations with bacteria. These associations are based on an exchange in which the bacterial symbiont provides functions that enhance host survival, and the host provides a stable, nutrient-rich home for the bacterial symbiont. Over the course of macroevolutionary time, the metabolic and physiological activities of the host and symbiont become increasingly integrated, leading to an obligate mutualism. Under these conditions, the host cannot survive without the ancillary functions provided by the bacterial symbiont and the bacterium cannot persist outside of the host. The reliance of the bacterial symbiont on the animal host is elegantly illustrated in a number of insect-bacterial symbioses. In these associations, dependency arises as a result of bacterial genome degeneration and size reduction [1]. In extreme cases, when the symbiotic partners have co-evolved for long periods of time, bacterial endosymbionts display pronounced genome streamlining. For example, bacterial endosymbionts of aphids, *Buchnera* spp., have genome sizes ranging from 0.42 to 0.66 Mbp, that are substantially smaller than those of close, free-living relatives (e.g. *Escherichia coli* K-12; 4.6 Mbp) [2].

Although genome streamlining is most conspicuous in ancient associations, the early stages of this process have been observed in symbiotic associations that are more recent in origin. For example, the relationship between tsetse flies and their bacterial endosymbiont *Sodalis glossinidius* is predicted to be relatively recent in origin [3], and although the genome size of *S. glossinidius* (4.29 Mbp) is similar to that of close, free-living relatives (e.g. *Yersinia enterocolitica*; 4.6 Mbp) [4], a significant portion of the *S. glossinidius* genome is composed of pseudogenes that have been inactivated as a consequence of relaxed selection, because they no longer play a vital role in the symbiosis [5]. However, despite this extensive genome degeneration, several recently derived endosymbionts (including *S. glossinidius*) have been shown to maintain intact copies of genes sharing high levels of sequence identity with homologs encoding virulence determinants in common plant and animal pathogens [6]–[10]. Since these virulence genes are not found in ancient mutualistic endosymbionts, it has been suggested that they play a transient role in the establishment of these symbiotic associations [1]. Notably, like many plant and animal pathogens, recently derived insect endosymbionts also maintain an extensive repertoire of regulatory genes [5], [11]. However, while pathogens use these regulators to rapidly coordinate adaptations to diverse environments (e.g. host vs. non-host), the mutualistic endosymbionts of insects are permanently host associated and are therefore entrenched in a more static lifestyle. The ecological changes associated with a lifestyle switch from opportunism to obligate host association are therefore predicted to mediate adaptive changes in the functions of regulatory circuits that serve as environmental sensors.

Many Gram-negative pathogens utilize the PhoP-PhoQ two-component regulatory system to modulate adaptive responses to changes in levels of divalent cations, including magnesium, in the environment [12]–[14]. When magnesium availability is high, the inner membrane sensor kinase PhoQ dephosphorylates the cytoplasmic response regulator PhoP, maintaining the system in a deactivated state. When magnesium availability is low, PhoQ autophosphorylates and transfers its phosphoryl group to PhoP. Phosphorylated PhoP then activates the expression of target genes that are associated with adaptation to the low magnesium environment [13], [14]. In multicellular eukaryotes, intra and extracellular concentrations of magnesium vary considerably. While extracellular concentrations are in the millimolar range, intracellular concentrations tend to be in the micromolar range [12], [13], [15]. Notably, in many facultative intracellular pathogens, PhoP-PhoQ regulates the expression of genes important for intracellular survival. Low intracellular magnesium levels drive PhoP-PhoQ-dependent expression of loci involved in magnesium transport [13], [14] and structural modifications of the lipid A portion of the bacterial lipopolysaccharide (LPS) [16]. Whereas magnesium transport genes allow bacteria to obtain adequate amounts of magnesium for survival, LPS modifications protect the bacteria against stressful conditions found within eukaryotic phagosomes, such as low pH and high levels of antimicrobial peptides (AMPs) [13], [15]. Notably, in addition to low magnesium, the PhoP-PhoQ system is also known to detect and respond to other host derived signals found within phagosomes, such as AMPs [17] and acidic pH [18]. In contrast to magnesium, which inhibits PhoP-PhoQ, the binding of AMPs or the exposure of cells to acidic conditions results in the activation of PhoP-PhoQ [17], [18].

At present, very little is known about how mutualistic endosymbionts evade or overcome the challenges imposed by the insect immune system. The immune systems of multicellular organisms utilize a vast array of mechanisms to combat invading microorganisms. The immune cells of both insects and vertebrates are known to synthesize various cationic AMPs that kill bacteria by interacting with lipid A and forming holes into the bacterial lipid membrane. In insects, these immune peptides combat bacterial pathogens by functioning as antibiotics that are secreted into the hemolymph and stored within phagocytic cells, where they are used to kill engulfed bacteria [19], [20]. To date, only two insect endosymbionts (*S. glossinidius* and *Candidatus* Arsenophonus arthropodicus) have been cultured in the laboratory and tested for resistance to cationic AMPs; notably, both of these endosymbionts were found to display high levels of resistance *in vitro* [21]–[23].

In the current study, we show that the mutualistic insect endosymbiont *S. glossinidius* utilizes a PhoP-PhoQ two-component regulatory system to modulate the expression of genes involved in lipid A modifications that confer bacterial resistance to host derived AMPs. In the absence of PhoP, *S. glossinidius* demonstrates increased sensitivity to host derived AMPs, an aberrant profile of type-III secretion system (TTSS) gene expression and an inability to colonize its natural host, the tsetse fly, and a close dipteran relative, the hippoboscid louse fly. In addition, our results indicate that the PhoP-PhoQ system of *S. glossinidius* has undergone sensory adaptations in the transition to a permanent association with its insect host.

## Results

### PhoP Is Required for Resistance to Cationic AMPs *In Vitro*

**Figure 1:**
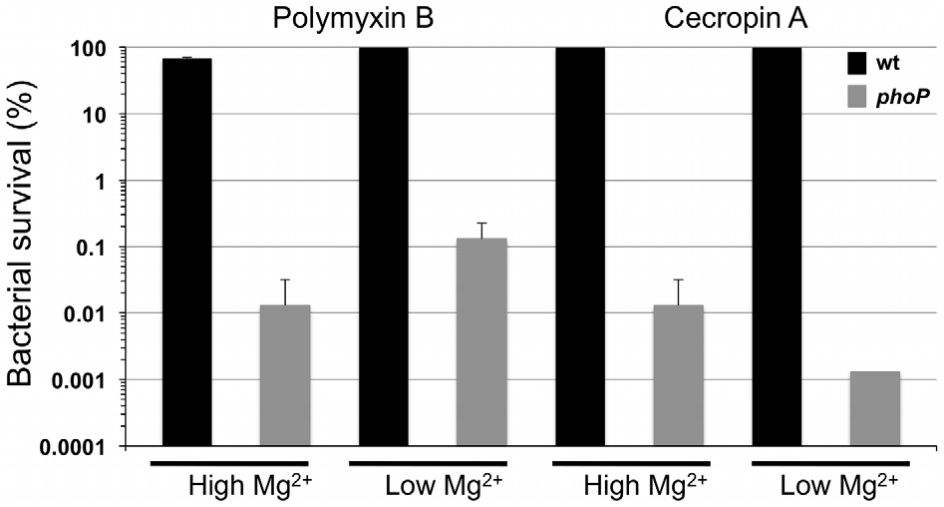
Resistance to polymyxin B and cecropin A is PhoP-dependent in *S. glossinidius.* Whereas magnesium has no effect on *S. glossinidius* wild type (wt) resistance to cecropin A, it has a small but significant, PhoP-independent effect on resistance to polymyxin B (two-tail t-test: p<0.01). Wild type and *phoP* mutant cells were cultured at high (10 mM) and low (10 µM) levels of magnesium, exposed to antimicrobial peptides and enumerated after plating. Error bars represent standard deviations.

In previous studies, *S. glossinidius* was shown to be highly resistant to the effects of a number of cationic AMPs [22], [23]. In the current study we examined the sensitivity of a *S. glossinidius phoP* mutant [24] to polymyxin B and the insect immune peptide, cecropin A, which is known to be produced by the host of *S. glossinidius* [25], [26]. Whereas wild type *S. glossinidius* demonstrated high levels of resistance as expected, the *phoP* mutant strain was found to be extremely sensitive (c. 1000-fold increase in sensitivity) to both AMPs (Figure 1). Resistance to polymyxin B, but not cecropin A, was slightly increased in response to low magnesium availability in the *S. glossinidius* culture medium (Figure 1). However, this effect was mediated in both the wild type and *phoP* mutant strains, indicating that it is PhoP-independent. These results show that PhoP-PhoQ plays a vital role in mediating AMP resistance in *S. glossinidius*.

### PhoP Activates Expression of a Gene Known to Mediate Resistance to Cationic AMPs

In facultative intracellular pathogens, resistance to AMPs is mediated by structural modifications of the lipid A portion of the bacterial LPS [12], [13]. Because cationic AMPs kill bacteria by interacting with unmodified lipid A and interfering with the permeability of the bacterial lipid membrane [17], [19], we reasoned that the *S. glossinidius phoP* mutant might lack the ability to carry out the necessary structural modifications of lipid A. Inspection of the *S. glossinidius* genome sequence revealed the presence of three loci (*pagP*, locus tag SG1577; *pmrE*, locus tag SG1368; and *pmrH*, the first gene in the *pmrHFIJKLM* operon, locus tags SG1845 to SG1839, respectively) encoding proteins known to mediate modifications that reduce the negative charge of lipid A, preventing the binding of positively charged AMPs [13]. PagP mediates the palmytoylation of lipid A, a structural modification associated with bacterial resistance to alpha-helical AMPs such as cecropin A. PmrE and the proteins encoded by the *pmrH* operon mediate the synthesis and incorporation of 4-aminoarabinose into the lipid A, a modification associated with bacterial resistance to the cyclic lipopeptide polymyxin B [27].

**Figure 2:**
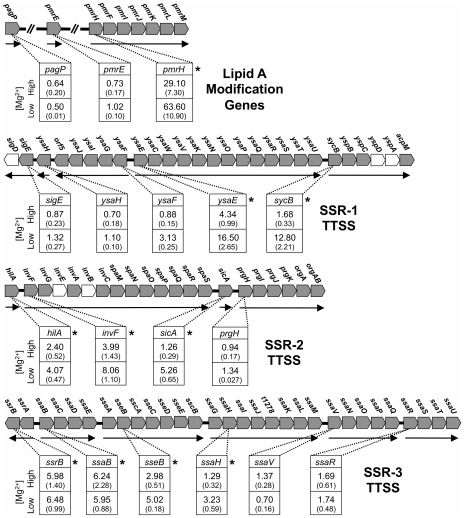
Quantitative PCR analysis of transcripts derived from genes involved in lipid A modifications in *S. glossinidius*. Genetic organization of lipid A modification genes is depicted: Functional genes are decorated in gray and putative transcriptional units are indicated by arrows. The numbers in boxes indicate the ratios of transcripts detected in the wild type strain relative to the *phoP* mutant strain of *S. glossinidius* grown under conditions of high (10 mM) and low (10 µM) magnesium availability. Genes displaying significant changes in expression in the wild type relative to the *phoP* mutant strain of *S. glossinidius* are highlighted with asterisks (two-tail t-test: p<0.05). Values in parentheses represent standard deviations.

Our quantitative PCR results show that *pagP* and *pmrE* are not regulated by PhoP (Figure 2). Instead, both genes were found to display high levels of expression under all conditions tested (data not shown), suggesting that they have likely evolved to a state of constitutive expression. However, the expression of the *pmrH* operon was found to be PhoP-dependent in *S. glossinidius* (Figure 2). When cells were grown in medium with high levels of magnesium, wild type *S. glossinidius* showed 29-fold higher levels of *pmrH* mRNA relative to the *phoP* mutant (Figure 2). Furthermore, when cells were grown in medium containing low levels of magnesium, the levels of *pmrH* mRNA were 63-fold higher in wild type *S. glossinidius* relative to the *phoP* mutant (Figure 2). Together, these results indicate that *S. glossinidius* uses the PhoP-PhoQ system to activate the transcription of the *pmrH* operon and direct modifications of lipid A that are important determinants of resistance to both polymyxin B and cecropin A.

### *Sodalis glossinidius phoP* Mutant Displays an Altered Lipid Composition

**Figure 3:**
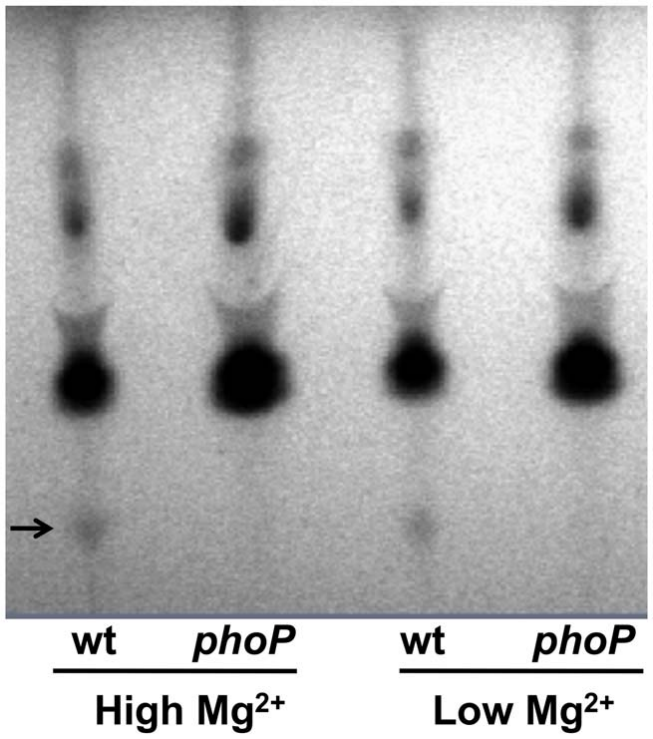
Thin layer chromatographic analysis of lipids extracted from wild type (wt) and *phoP* mutant strains of *S. glossinidius* grown at high (10 mM) and low (10 µM) concentrations of magnesium. Arrow highlights a lipid species that is absent in the *phoP* mutant strain.

Because PhoP-PhoQ often govern the expression of genes involved in the structural modifications of the outer surface of the bacterial lipid membrane [16], [28] we sought to determine if the genetic inactivation of *phoP* resulted in changes in the overall lipid composition of *S. glossinidius* cells. We carried out a thin layer chromatography (TLC) analysis of lipids derived from the wild type and *phoP* mutant strains of *S. glossinidius*. Our TLC analysis shows that the wild type *S. glossinidius* produces an additional (as yet uncharacterized) lipid species that is absent in the *phoP* mutant strain (Figure 3). This result reinforces the notion that *S. glossinidius* also utilizes the PhoP-PhoQ system to regulate expression of genes involved in lipid metabolism.

### PhoP Also Regulates Type-III Secretion System Genes in *Sodalis glossinidius*

**Figure 4:**
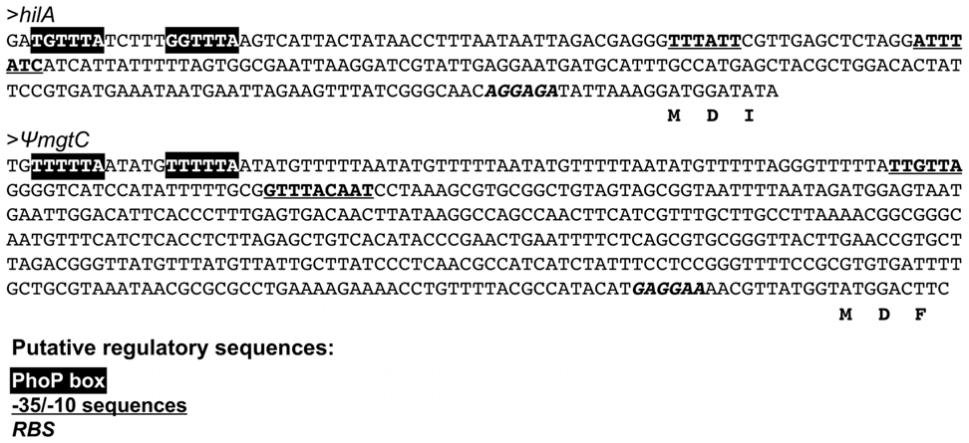
The putative promoter regions of the *S. glossinidius hilA* homologue and the *mgtCB* pseudo-operon contain canonical PhoP boxes. PhoP boxes (inverted text) and putative ribosomal binding site (bold) were identified by visual inspection of the promoter regions. Putative -35 and -10 regions (underlined) were identified using the online BPROM tool (SoftBerry, Inc.).

Some PhoP regulated promoters have a characteristic direct repeat sequence, known as a PhoP box, which serves as a binding site for phosphorylated PhoP [29], [30]. Inspection of the *S. glossinidius* genome sequence revealed the presence of a consensus PhoP box upstream of *hilA* (accession no. AAS66857; Figure 4), which is known to encode a master regulator of type-III secretion in *Salmonella enterica* [31]. Because the *S. glossinidius* genome has three distinct symbiosis regions (designated *Sodalis* Symbiosis Regions; SSR's) encoding TTSS genes [5], we elected to measure the effect of *phoP* inactivation on the basal expression levels of the genes found in these three distinct chromosomal locations using quantitative PCR. The *phoP* mutant was found to have significantly lower levels of transcripts encoding YsaE, SycB (SSR-1), HilA, InvF, SicA (SSR-2), SsrB, SsaB, SseB and SsaH (SSR-3; Figure 2). These results indicate that the *S. glossinidius* PhoP-PhoQ two-component system is also involved in the activation of TTSS genes that are known to be required for the invasion of insect cells and for intracellular survival [8].

### *Sodalis glossinidius phoP* Mutant Fails to Superinfect Insect Hosts

One important component of insect immunity involves the synthesis and secretion of high quantities of AMPs into the hemolymph [20]. Since insect endosymbionts, including *S. glossinidius*, are often found in the hemolymph of their insect hosts [32], [33], and because *S. glossinidius* uses TTSSs to invade and replicate within insect cells [6], [8], we assessed the ability of the *phoP* mutant strain of *S. glossinidius* to superinfect their natural tsetse fly host, *Glossina morsitans morsitans*, and a closely-related hippoboscid louse fly, *Pseudolynchia canariensis*, following intrathoracic microinjection. The superinfection approach [34] was selected to avoid the requirement to treat host insects with antibiotics to remove native symbionts, because this procedure is known to compromise the immune system of the fly [35]. The presence of native *S. glossinidius* in the tsetse fly therefore mandated the use of a recombinant (*fliM* mutant) *S. glossinidius* strain in lieu of a wild type control strain so that microinjected bacteria could be differentiated from native symbionts by PCR. In comparison to the *fliM* mutant strain, the *phoP* mutant strain was found to be completely defective in its ability to superinfect the host insect (Table 1). At seven days following microinjection, we were able to detect the *fliM* mutant strain in 26 out of 32 tsetse flies sampled. In contrast, we were unable to detect either the *phoP* or *fliM* heat-killed mutant strains in any of the tsetse flies sampled at seven days post-microinjection (Table 1). In the hippoboscid louse flies, which are closely related to tsetse flies [36] but do not maintain a native population of *S. glossinidius*, we compared the superinfection abilities of wild type, *fliM* and *phoP* mutant strains of *S. glossinidius*. In this experiment, wild type and *fliM* mutant *S. glossinidius* were detected in all flies sampled at seven days following microinjection, and the *phoP* mutant and heat-killed strains of *S. glossinidius* were not detected in any flies at seven days post injection (Table 1). Based on these results, we conclude that the *phoP* mutant strain of *S. glossinidius* is killed by the insect immune system following superinfection in both tsetse flies and hippoboscid louse flies. This indicates that the PhoP-PhoQ two-component regulatory system is essential for the establishment and maintenance of an *S. glossinidius* infection in an insect host.

**Table 1:** PCR detection of *Sodalis glossinidius* seven days following microinjection in tsetse flies and louse flies.

*S. glossinidius* strain	Number of PCR positive samples/number of total samples	
	Tsetse flies	Louse flies
**Wild-type**	N.D.	19/20
**Wild-type heat-killed**	N.D.	0/20
***fliM***	26/32	16/16
***fliM*** ** heat-killed**	0/32	N.D.
***phoP***	0/32	0/20

Differences in colonization patterns between the wild-type, *fliM* and *phoP* strains are statistically significant (Pearson X^2^ test, p<0.0001). “N.D.” indicates that that a particular experiment was not performed.

### Effects of Acidic pH, Magnesium, and Cationic AMPs on *Sodalis glossinidius*

In many facultative bacterial pathogens, the PhoP-PhoQ system functions as a magnesium sensor that controls the expression of genes mediating physiological adaptations to changes in environmental levels of magnesium [13]-[15]. In addition, the PhoQ sensor kinase is known to detect and respond to conditions of acidic pH [18], and the binding of cationic AMPs that displace magnesium ions [17]. Several lines of evidence indicate that the *S. glossinidius* PhoP-PhoQ system has a diminished ability to respond to environmental magnesium. First, in *S. enterica* AMP resistance is controlled by the PhoP-PhoQ two-component system. *Salmonella enterica* cells grown under conditions of high magnesium availability are >1000-fold more susceptible to AMPs [37]. In contrast, our AMP resistance assay showed that although PhoP-PhoQ controls AMP resistance in *S. glossinidius,* this phenotype is only marginally affected by magnesium availability in the culture medium (Figure 1). Second, our gene expression analyses show that PhoP activates the transcription of *pmrH* and a number of TTSS genes in *S. glossinidius*. However, while the expression of some of these genes is considerably higher in wild type *S. glossinidius* relative to the *phoP* mutant strain, transcriptional changes in response to environmental magnesium were unexpectedly small. Finally, the results from our TLC lipid analysis showed that, regardless of magnesium concentration, wild type *S. glossinidius* synthesizes a lipid species that is not found in the *phoP* mutant strain (Figure 3).

**Figure 5:**
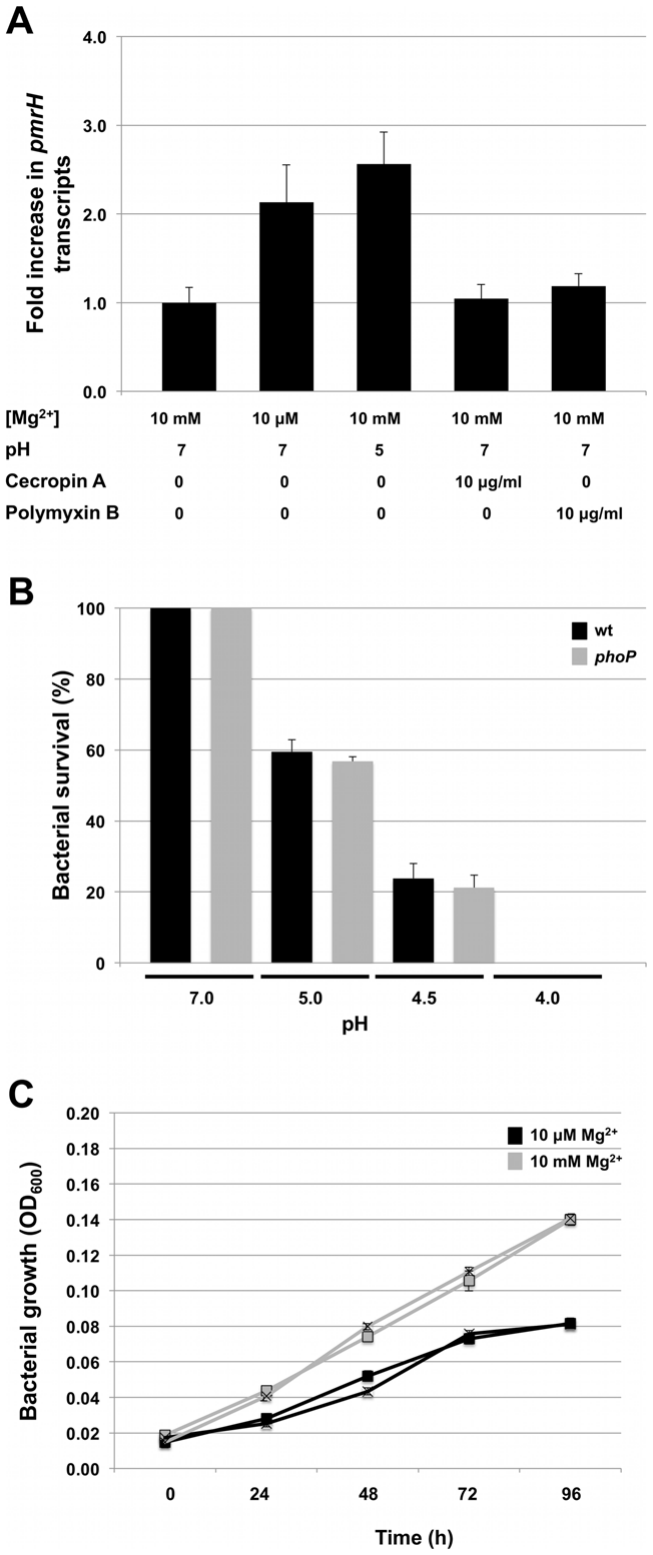
Response of *S. glossinidius* to antimicrobial peptides, acidic pH, or magnesium. A. Quantitative PCR analysis of *pmrH* transcripts derived from *S. glossinidius* cells grown under various medium conditions. Fold increase in *pmrH* transcripts was calculated relative to cells grown in defined medium containing 10 mM of magnesium at pH 7. B. Percent survival of *S. glossinidius* wild type and *phoP* mutant cells at various pH values. C. Growth dynamics of *S. glossinidius* wild type (▪) and *phoP* mutant (×) in defined medium containing high (10 mM) and low (10 µM) concentrations of magnesium. Error bars represent standard deviations.

To further explore the role of magnesium, acidic pH and the presence of AMPs on signaling mediated by the *S. glossinidius* PhoQ, we first used quantitative PCR to measure changes in the numbers of transcripts of *pmrH* in wild type cells under different culture conditions. The results show that *pmrH* expression was only slightly increased under conditions of low magnesium availability or low pH (2-fold and 2.6-fold increases, respectively). Furthermore, the presence of the AMPs cecropin A or polymyxin B in the culture medium had no significant effect on *pmrH* gene expression (Figure 5A).

Since cellular adaptations mediated by PhoP-PhoQ are anticipated to be functionally linked to the physiological signals sensed by PhoQ, we also tested the abilities of wild type and *phoP* mutant strains of *S. glossinidius* to resist acid stress, and to grow under conditions of magnesium starvation. Notably, both wild type and *phoP* mutant strains of *S. glossinidius* showed the same level of sensitivity towards acid stress (Figure 5B). Similarly, no significant difference was observed in the abilities of wild type and *phoP* mutant strains of *S. glossinidius* to grow in a defined medium in which bacterial growth was clearly limited by a lack of magnesium (Figure 5C). Together, these results indicate that (i) the *S. glossinidius* PhoP-PhoQ system displays an atypical, diminished response to magnesium, acidic pH and AMPs, and (ii) the *S. glossinidius* PhoP response regulator does not facilitate tolerance towards acid stress or magnesium starvation in *S. glossinidius*, as it does in several enteric pathogens.

### *Salmonella enterica* Strains Expressing *Sodalis glossinidius* PhoQ Are Magnesium-Insensitive

To further analyze the magnesium sensing capability of the *S. glossinidius* PhoQ sensor kinase we took advantage of an approach that was initially devised to study the magnesium binding property of the *S. enterica* PhoQ homologue [38]. We utilized two *S. enterica* strains (EG5931 and EG9461) both of which encode a *phoQ* null allele and a transcriptional fusion of the lac gene to a chromosomal copy of the *S. enterica* PhoP activated *pmrC* gene (*pmrC*::MudJ). Whereas one of these strains (EG9461) has a wild type *phoP* allele and can be used to measure induction levels of PhoP-activated genes at low magnesium availability, the other strain (EG5931) has a phoP* allele encoding a protein that efficiently autophosphorylates from acetyl phosphate (i.e., independently from PhoQ). Because PhoP* responds normally to the phosphatase activity of PhoQ, strain EG5931 can be used to examine the capability of PhoQ to dephosphorylate PhoP and repress transcription of PhoP-activated genes [38], [39].

**Figure 6:**
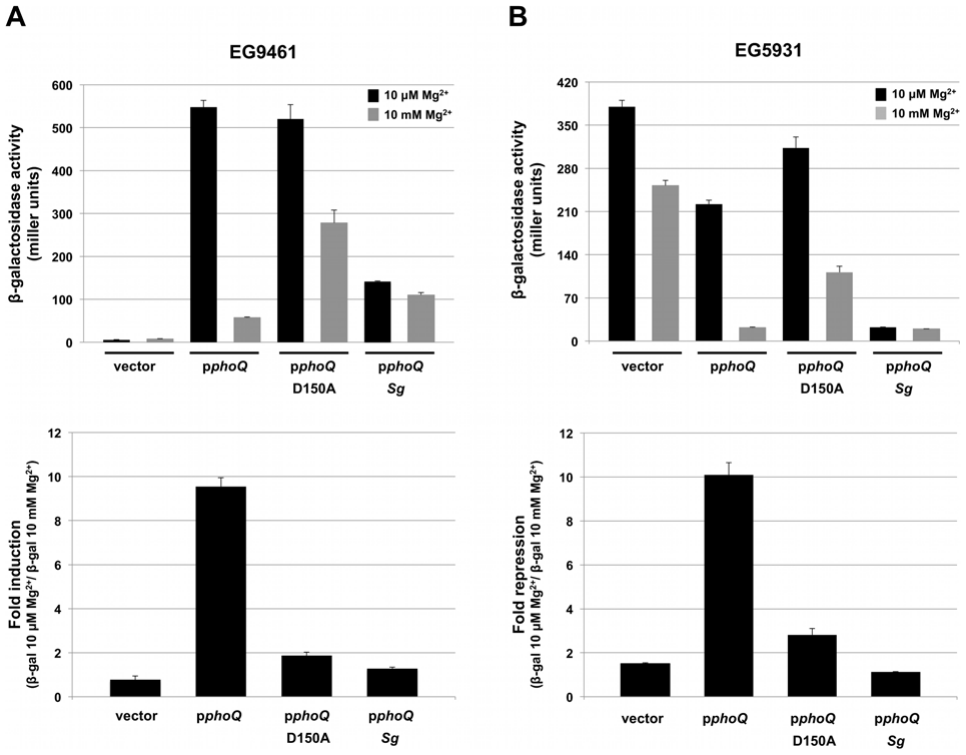
*Salmonella enterica* strains expressing *S. glossinidius* PhoQ do not respond to magnesium. A. β-galactosidase activity (top) of the *S. enterica* PhoP-activated *pmrC* gene produced by strain EG9461 (*phoQ*::Tn*10 pmrC*:: MudJ) harboring the plasmid pUHE21-2 lacI^q^ (vector) or pUHE21-2 lacI^q^ derivatives expressing the *S. enterica* PhoQ (p*phoQ*), *S. enterica* PhoQ variant with mutation D150A (p*phoQ* D150A) or *S. glossinidius* PhoQ (p*phoQ Sg*). Fold induction (bottom) of *pmrC*::MudJ β-galactosidase activity produced by strain EG9461 harboring various plasmid constructs in response to magnesium. B. β-galactosidase activity (top) of the *S. enterica* PhoP-activated *pmrC* gene produced by strain EG5931 (phoP* *phoQ*::Tn*10 pmrC*:: MudJ) harboring various plasmid constructs. EG5931 encodes a *phoP* allele (phoP*) that efficiently phosphorylates from acetyl phosphate (i.e., independently of PhoQ) [39]. Fold repression (bottom) of *pmrC*::MudJ β-galactosidase activity produced by strain EG5931 harboring various plasmid constructs in response to magnesium. Error bars represent standard deviations.

We used strain EG9461 to test the ability of the *S. glossinidius* PhoQ sensor kinase to induce the expression of PhoP-activated genes in response to low magnesium availability. For controls, we complemented strain EG9461 with either a plasmid vector alone, a plasmid expressing the *S. enterica* wild type PhoQ sensor kinase (p*phoQ*) or a plasmid expressing a *S. enterica* PhoQ variant with mutation D150A (p*phoQ* D150A) in the magnesium ligand-binding site [38] (Figure S1). Strain EG9461 harboring the plasmid vector alone does not express a PhoQ protein and, therefore, produces no β-galactosidase activity from the *pmrC*::MudJ reporter fusion (Figure 6A). On the other hand, when this strain is complemented with a plasmid expressing the *S. enterica* wild type PhoQ sensor kinase (p*phoQ*), the expression of the reporter fusion mimics that of wild type cells; this strain displays high expression at low magnesium levels and low expression at high magnesium levels (Figure 6A). When complemented with a plasmid expressing the *S. enterica* PhoQ variant with mutation D150A (p*phoQ* D150A), strain EG9461 is derepressed under conditions of high magnesium availability (Figure 6A) [38]. In addition, when strain EG9461 is complemented with a plasmid expressing the *S. glossinidius* PhoQ protein (p*phoQ Sg*) there is an increase in β-galactosidase activity of the reporter fusion relative to that of strain EG9461 harboring the plasmid vector alone (Figure 6A), indicating that *S. glossinidius* PhoQ does phosphorylate the *S. enterica* PhoP response regulator. However, consistent with our previous observation, the resulting strain shows only a slight change in reporter gene activity in response to magnesium (Figure 6A).

Similar results were obtained in an experiment involving *S. enterica* strain EG5931, which expresses an autophosphorylating phoP* allele. In this case strain EG5931, harboring the plasmid vector alone, displayed high levels of β-galactosidase activity from the reporter fusion, regardless of magnesium availability (Figure 6B). Yet, because the phoP* allele responds normally to the phosphatase activity of PhoQ, complementation of strain EG5931 with a plasmid expressing the *S. enterica* wild type PhoQ (p*phoQ*) produced a strain that displayed a gene expression profile mimicking that of wild type cells. As with strain EG9461, complementation of strain EG5931 with a plasmid expressing the *S. enterica* PhoQ variant with mutation D150A (p*phoQ* D150A) gave rise to a strain that had a reduced capability to promote magnesium mediated repression of the *pmrC*::MudJ reporter fusion (Figure 6B) [38]. In addition, when strain EG5931 was complemented with a plasmid expressing the *S. glossinidius* wild type PhoQ protein (p*phoQ Sg*), the resulting strain again demonstrated little or no response to magnesium. Furthermore, this strain demonstrated decreased β-galactosidase activity of the reporter fusion relative to that of strain EG5931 harboring the plasmid vector alone (Figure 6B), indicating that the *S. glossinidius* wild type PhoQ sensor kinase does maintain a phosphatase activity. Together, these results further support our observation that the *S. glossinidius* PhoQ has a substantially diminished ability to sense magnesium.

### Evidence for Magnesium Sensing in an Ancestral Precursor of the *S. glossinidius* PhoQ

Given that the majority of PhoP-PhoQ homologues studied in bacteria are known to respond aggressively to changes in acidic pH, magnesium and cationic AMPs [13], [14], [17], [18], it is striking that the *S. glossinidius* PhoQ sensor kinase displays a substantially reduced ability to respond to these signals. In many facultative pathogens, PhoP-PhoQ is known to play an important role in mediating magnesium homeostasis [13], [14]. When PhoQ senses conditions of low magnesium availability, PhoP responds by increasing expression of specialized, ATP-driven magnesium transporters (designated MgtA and MgtB in *S. enterica*) [14], [40], [41]. While *S. glossinidius* maintains intact copies of the generalized magnesium transporters *corA* (locus tag SG2341) and *mgtE* (locus tags SG0628 and SG1738) that are not regulated by PhoP-PhoQ in other bacteria, it completely lacks *mgtA* and maintains only a disrupted copy of *mgtB* with multiple frameshifts (Table 2). However, the disrupted copy of *mgtB*, encoded within the *mgtCB* pseudo-polycistron maintains a canonical PhoP box (Figure 4), implying that in the recent evolutionary past *S. glossinidius* used PhoP-PhoQ to coordinate the expression of genes involved in magnesium transport. Since it would be unexpected to have a magnesium transport system controlled by a PhoP-PhoQ system that is unable to sense magnesium, we conclude that an ancestral precursor of the *S. glossinidius* PhoQ protein possessed an increased magnesium sensing capability. This implies that the reduction in *S. glossinidius* PhoQ's ability to sense magnesium is a derived characteristic.

**Table 2:** Distribution of *phoP*-*phoQ*, the magnesium transporters *mgtA* and *mgtB*, and lipid A modification genes among the insect pathogen *Photorhabdus luminescens* and recently derived and ancient insect endosymbionts.

Organism	Classification	Genome size (Mbp)	Age of Association	*phoP*	*phoQ*	*mgtA*	*mgtB*	Lipid A modification genes								
								*pagP*	*pmrE*	*pmrF*	*pmrH*	*pmrI*	*pmrJ*	*pmrK*	*pmrL*	*pmrM*
*Photorhabdus luminescens*	Insect pathogen	5.68	N. A.	**+**	**4**	**−**	**P**	**+**	**+**	**+**	**+**	**+**	**+**	**+**	**+**	**+**
*Arsenophonus nasoniae*	Insect symbiont	>3.56	Unknown	**+**	**2**	**−**	**−**	**+**	**+**	**+**	**+**	**+**	**+**	**+**	**+**	**+**
*Sodalis glossinidius*	Insect symbiont	4.17	Unknown	**+**	**2**	**−**	**P**	**+**	**+**	**+**	**+**	**+**	**+**	**+**	**+**	**+**
*Candidatus* Hamiltonella defensa	Insect symbiont	2.10	Unknown	**−**	**−**	**+**	**−**	**−**	**+**	**+**	**−**	**+**	**+**	**+**	**+**	**+**
*Candidatus* Serratia symbiotica	Insect symbiont	2.57	Unknown	**+**	**1**	**−**	**−**	**−**	**+**	**+**	**−**	**−**	**−**	**+**	**+**	**−**
*Wigglesworthia glossinidia*	Insect symbiont	0.69	40**−**80	**−**	**−**	**−**	**−**	**−**	**+**	**−**	**−**	**+**	**+**	**+**	**−**	**−**
*Blochmannia* spp.	Insect symbiont	0.70–0.79	50	**−**	**−**	**−**	**−**	**-**	**−**	**−**	**−**	**−**	**−**	**−**	**−**	**−**
*Carsonella ruddii*	Insect symbiont	0.16	120	**−**	**−**	**−**	**−**	**−**	**−**	**−**	**−**	**−**	**−**	**−**	**−**	**−**
*Buchnera*spp.	Insect symbiont	0.42–0.65	150	**−**	**−**	**−**	**−**	**−**	**−**	**−**	**−**	**−**	**−**	**−**	**−**	**−**
*Baumannia cicadellinicola*	Insect symbiont	0.68	170	**−**	**−**	**−**	**−**	**−**	**−**	**−**	**−**	**−**	**−**	**−**	**−**	**−**

Estimated age of association (in million of years) is shown for ancient insect symbionts [58]. “P” indicates that a given sequence was determined to be a pseudogene. ”N. A.” signifies not applicable. Numbers of acidic residues within the acidic patch are displayed for PhoQ homologues. Note also that *Candidatus* Serratia symbiotica maintains a truncated PhoQ homologue.

## Discussion

The transition in lifestyle from opportunism to obligate host association is often accompanied by the inactivation and loss of genes that are assumed to have played an important role in a facultative lifestyle but no longer provide an adaptive benefit in an obligate host-associated lifestyle [1]. While these degenerative changes are not expected to negatively impact the function of the symbiotic relationship, they are anticipated to increase host dependence as a result of niche specialization on the part of the symbiont. Furthermore, the transition from a dynamic lifestyle to a static, obligate host-associated lifestyle is expected to reduce the requirement for symbionts to engage in regulatory activities that normally enhance the ability of bacteria to survive in a changing environment.

In the current study we explored the role of the two-component regulatory system, PhoP-PhoQ, in the insect endosymbiont, *S. glossinidius*, which is an obligate, mutualistic associate of tsetse flies [42]. PhoP-PhoQ is of particular interest because it is found in a wide range of facultative pathogens [13], [14], [16] and plays an important role in enabling these bacteria to sense their presence in the host environment and mediate changes in gene expression that facilitate important adaptations to the host-associated lifestyle. Our results show that PhoP-PhoQ also plays an essential role in the maintenance of the mutualistic association between *S. glossinidius* and its insect host. In the absence of *phoP*, *S. glossinidius* is highly sensitive to the bactericidal effects of AMPs *in vitro* and is incapable of superinfecting either its natural host or a closely related hippoboscid louse fly.

In *S. enterica* and other facultative pathogens, the sensor kinase PhoQ plays an important role in the sensing of magnesium, AMPs and acidic pH. Thus, the most striking result to emerge from the current study is that the PhoP-PhoQ system of *S. glossinidius* differs from its counterparts in other bacteria by having a substantially reduced ability to sense these environmental cues. For example, the *S. glossinidius* PhoP-PhoQ system elicited only a minor reduction (c. 2-fold) in the expression of target genes in response to a 1000-fold increase in the level of magnesium in the culture medium. Similarly, the *S. glossinidius* PhoP-PhoQ system was found to be largely unresponsive to a change in pH; *S. glossinidius* cells cultured under acidic conditions showed only a 2.6-fold increase in the expression of a PhoP-regulated target gene relative to cells cultured at neutral pH. In *S. enterica*, the constitutive expression of PhoP-activated genes is known to have a deleterious impact on bacterial virulence and survival in host tissues [43], due to the fact that the initial activation of PhoP induces a transcriptional surge that enables *S. enterica* to rapidly initiate virulence gene expression [44]. In the current study, we were unable to identify any conditions under which the *S. glossinidius* PhoP-PhoQ system was effectively repressed. *In vitro*, *S. glossinidius* was found to display high levels of resistance to AMPs under all conditions tested, indicating that the dynamics of PhoP-based gene regulation in *S. glossinidius* are locked in a constitutively active state, at least with respect to signaling mediated by magnesium, AMPs and pH change.

The presence of a canonical PhoP binding site in the promoter sequence of an inactivated *mgtB* allele indicates that in the recent evolutionary past, *S. glossinidius* used a magnesium-sensing PhoP-PhoQ to control the expression of a high affinity magnesium transporter. The proposed reduction in PhoQ's ability to sense magnesium (and other signals) can be rationalized in several ways. First, if the primary mandate of PhoQ's magnesium sensing capability was to control magnesium uptake, then the inactivation of the gene encoding this magnesium transporter could have facilitated relaxed selection on the ability of PhoQ to sense magnesium. Second, since magnesium binding drives the repression of PhoP-regulated target genes, the loss of a requirement to repress these genes, perhaps resulting from a switch to a static host-associated lifestyle, could also have facilitated relaxed selection on PhoQ's sensing capabilities. Third, it is conceivable that the loss of PhoQ's ability to sense magnesium and acidic pH provided an adaptive advantage in *S. glossinidius*' current lifestyle. Like many recently derived insect endosymbionts, *S. glossinidius* inhabits the hemolymph of its insect host [32] where it is anticipated to be exposed simultaneously to high concentrations of both AMPs [20], [25], [26] and magnesium [12], [45], at neutral pH [46]. In this environment, the magnesium- or pH-mediated repression of PhoP-regulated genes would likely be deleterious due to the fact that it would yield an AMP sensitive phenotype that is incompatible with survival in the AMP-rich hemolymph. Conversely, a magnesium-insensitive PhoQ would be expected to facilitate the constitutive activation of PhoP-regulated target genes, ensuring resistance towards AMPs in magnesium-rich hemolymph.

Since the mechanism of magnesium binding by the *S. enterica* PhoQ homologue has been well characterized through structural and functional studies [38], [47], we inspected the sequence of the *S. glossinidius* PhoQ protein to determine if there are any obvious modifications that can explain it's reduced sensitivity to magnesium. Notably, the *S. glossinidius* PhoQ sequence maintains several amino acid substitutions that replace key acidic residues in a location corresponding to a magnesium ligand-binding site in the PhoQ protein of *S. enterica* (Figure S1) [38], [47]. In *S. enterica*, mutant strains harboring PhoQ sequences lacking just one of these key acidic residues demonstrate >5-fold reduction in their ability to repress the transcription of PhoP-activated genes in response to magnesium (p*phoQ* D150A in Figure S1 and [38]). Thus it is conceivable that the loss of three key acidic residues in the *S. glossinidius* PhoQ substantially reduced the ability of this sensor kinase to bind magnesium, explaining the reduction in the magnesium dependent PhoQ transcriptional repression of PhoP-activated genes observed in *S. glossinidius*. An alignment of PhoQ sequences derived from a selected range of gamma Proteobacteria shows that PhoQ homologues from two insect endosymbionts (*S. glossinidius* and *Arsenophonus nasoniae*) along with the soft-rot plant pathogen *Dickeya dadantii* (formerly *Erwinia chrysanthemi*) each have putative magnesium-binding patches that lack the requisite acidic residues and are therefore predicted to be compromised in terms of magnesium-sensing (Figure S1A). While mutations in this conserved region likely contribute to a decreased magnesium sensing capability, it is possible that changes in other regions of PhoQ also contribute to this phenotype. Indeed, we noticed that the *S. glossinidius* PhoQ homologue also maintains a hydrophobic phenylalanine residue located at position 121, instead of a charged histidine residue that is found in the *S. enterica* PhoQ (Figure S1A). In *S. enterica*, this charged residue has also been shown to be important for magnesium binding and magnesium mediated repression [38].

Although magnesium sensing is recognized as the primary function of PhoQ [14], [15], we cannot rule out the possibility that the *S. glossinidius* PhoQ has evolved the ability to sense a novel signal in the insect host. For example, it has recently been shown that the PhoQ protein of *Edwardsiella tarda* has evolved to detect changes in temperature, in addition to magnesium, to coordinate the expression of protein secretion systems that play an important role in virulence [48]. However, it is clear that the *S. glossinidius* PhoQ has lost the ability to sense key signals that play an important role in the functionality of PhoP-PhoQ in many pathogens. The adaptive benefit of PhoP-PhoQ in *S. glossinidius* now appears to be solely due to the ability of this regulatory system to serve as a constitutive driver for the expression of genes that have critical functions in the symbiosis. To this end, the modification or loss of sensory functions in a two-component system is not necessarily paradoxical, as long as an adaptive benefit is realized as a result of the output of the response regulator. Of course a similar functional outcome could be achieved by modulating the promoter sequences of all PhoP-regulated genes to achieve constitutive expression in the absence of PhoP. Although more evolutionary steps are needed to achieve this outcome, it might ultimately be favored by natural selection because it represents a more frugal solution. Indeed, such a transition might already be underway in *S. glossinidius*, evidenced by the fact that the lipid A modification genes, *pagP* and *pmrE,* are expressed independently of PhoP, whereas PhoP is required for the expression of these genes in *S. enterica* [27].

In a wider evolutionary context, it is interesting to note that intact homologues of *phoP-phoQ* and the lipid modification genes are only present in the genome sequences of recently established insect endosymbionts (Table 2). This suggests that the functions of PhoP-PhoQ and the lipid A modification genes are required only as a stopgap in the early stages of symbiotic interactions, to enable bacteria to resist attack from the host immune system to facilitate the establishment and maintenance of persistent infections in host tissues. This is supported by the results of our superinfection experiments, which show that a *phoP* mutant strain of *S. glossinidius* is unable to establish infection in an insect host. Taken together, these observations suggest that recently established insect endosymbionts have an intrinsic ability to overcome the insect immune system and establish persistent infections in insects, and this may help to explain the broad distribution of certain recently established insect endosymbionts (e.g. relatives of *S. glossinidius* and *Arsenophonus* spp.) in distantly related host insects [10], [21], [42], [49]–[51]. It also explains differences observed in patterns of co-evolution between insects and endosymbionts of different inferred ages. While the phylogenies of insects and their ancient endosymbionts demonstrate high levels of congruence, implying little or no ongoing horizontal symbiont transmission or novel colonization events, the phylogenies of insects and their recently established endosymbionts are often discordant [1].

At a broad level, the results obtained in the current study reinforce the notion that the molecular mechanisms facilitating host-symbiont interactions have a common origin in both pathogenic and mutualistic associations. More specifically, our findings illustrate the capability of a complex regulatory circuit to adapt to a change in lifestyle. Furthermore, the degeneration of the sensing capability of PhoQ may represent a snapshot of a wider picture of regulatory simplification that is concomitant with a transition to a static, mutualistic, host-associated lifestyle that reduces the requirement for bacteria to engage in environmental sensing.

## Materials and Methods

### Bacterial Strains and Culture Conditions

*Escherichia coli* W25113 was maintained in Luria-Bertani (LB) medium at 37°C, or 30°C when harboring the repA101 (ts) plasmid pKD46 [52]. *Salmonella enterica* strains were maintained at 37°C in either LB medium or N-minimal medium [38] containing either 10 mM or 10 µM MgCl_2_. *Sodalis glossinidius* strains were maintained at 25°C in the semi-defined liquid Mitsuhashi- Maramorosch (MM) medium as described previously [42] or in a defined medium composed of 6 g/l casamino acids, 4 g/l glucose, 0.2 g/l KCl, 7.0 g/l NaCl, 0.12 g/l NaHCO_3_, 0.18 g/l NaH_2_PO_3_, 10 µg/ml of thiamine, 10 mM or 10 µM of CaCl_2_, and 10 mM or 10 µM MgCl_2_, pH 7. When rapidly dividing symbiont cultures were needed, liquid cultures of *S. glossinidius* were maintained in an orbital shaker at 200 rpm. For isolation of symbiont clones, bacteria were plated on 1% MM agar plates, supplemented with 5% defibrinated horse blood, and incubated at 25°C under microaerophilic conditions (5% Oxygen, 10% CO_2_, balanced with N_2_). Where appropriate, antibiotics were added to culture media at the following concentrations: 100 µg/ml (*E. coli* and *S. enterica*) or 50 µg/ml (*S. glossinidius*) of ampicillin, 15 µg/ml (chromosomal insertions) or 50 µg/ml (high copy number plasmids) of chloramphenicol, 50 µg/ml of kanamycin, 10 µg/ml tetracycline. When needed, 3′-5′-cyclic adenosine monophosphate (cAMP; Sigma Aldrich) was added to the culture medium at a final concentration of 5 mM.

### Construction of Replacement Alleles

Replacement alleles for *S. glossinidius* were generated using the lambda-Red recombineering system in *E. coli* [52]. The strategy for construction of replacement alleles for *S. glossinidius* is illustrated in Figure S2. Briefly, *S. glossinidius* genes were amplified by polymerase chain reaction (PCR) using Phusion High-Fidelity DNA polymerase (New England Biolabs). PCR products were ligated into pCR-Blunt II-TOPO (Invitrogen) and transformed into *E. coli* by electroporation. Clones were grown and plasmid DNA was isolated using the QIAprep Spin Miniprep Kit (Qiagen). Recombinant plasmids were then transformed into *E. coli* (pKD46) and replacement alleles were generated by lambda-Red mediated insertion of a chloramphenicol resistance cassette (derived from pEpiFOS-5, Epicentre), according to the method described previously [52]. Following curing of pKD46, plasmids containing the replacement alleles were extracted, diluted and introduced into *E. coli* by electroporation. The replacement alleles were verified by DNA sequencing and plasmids containing the correct allele sequence were used as PCR templates for the generation of linear DNA substrates for recombineering in *S. glossinidius*.

### Transformation of *Sodalis glossinidius*

All *S. glossinidius* transformations (using both plasmid and linear DNA constructs) were conducted using a heat-shock method [53]. Following transformation, the cells were allowed to recover overnight at 25 °C in liquid MM medium prior to plating.

### Lambda-Red Mediated Insertions in *Sodalis glossinidius*

Lambda-Red mediated chromosomals insertions were generated as described previously [24]. Briefly, cultures of *S. glossinidius* harboring pKD46 were grown without shaking to an OD_600nm_ of approximately 0.2. The cultures were then transferred to a shaking incubator and grown overnight until OD_600nm_ reached 0.5. The cells were pelleted at 5,000 × *g* for 10 min at 4°C, and washed twice with an equal volume of 0.85% (w/v) NaCl and resuspended in a final volume of 1 ml of 0.85% (w/v) NaCl. The cell suspensions were inoculated into 200 ml of fresh MM liquid medium [42] supplemented with 0.5% (w/v) arabinose and 5 mM cAMP, and incubated at 25°C with shaking for 30 min to facilitate induction of the lambda-Red functions. After induction, the cells were made chemically competent and transformed with linear DNA using the heat-shock method [53]. Following overnight recovery, the cells were resuspended in 150 µl of MM liquid medium. To assess cell viability following transformation, 15 µl of cell suspension was plated on MM blood agar plates alone. To select for recombinant clones, the remaining 135 µl was spread on an MM blood agar plate containing ampicillin and chloramphenicol. Plates were incubated under microaerophilic conditions and inspected for growth after 9 days. Putative recombinant clones were then isolated as single colonies and the presence of chromosomal insertions was confirmed by DNA sequencing.

### Curing of Lambda-Red Plasmid from *Sodalis glossinidius*

Following lambda-Red recombineering, plasmid pKD46 was cured from *S. glossinidius* by maintaining recombinant strains in the absence of plasmid selection. Cultures were grown under these conditions with shaking for approximately 50 generations prior to passage into fresh medium. Cultures were passaged a total of five times and then plated on MM blood agar plates supplemented with chloramphenicol alone. After 7 days of growth, colonies were screened for ampicillin sensitivity by replica plating onto MM blood agar plates supplemented with chloramphenicol and ampicillin.

### Antimicrobial Peptide Resistance Assay

Antimicrobial peptide resistance assays were performed using a modified version of a previously described method [54]. Wild type and *phoP* mutant strains of *S. glossinidius* were grown to mid-log phase in MM liquid medium. 20 ml of each culture was transferred to 50 ml tubes and the cells were harvested by centrifugation at 5,000 × *g* for 10 minutes at 4°C. The cells were washed twice with an equal volume of 0.85% (w/v) NaCl and resuspended in 1 ml of 0.85% (w/v) NaCl. 0.5 ml of each cell suspension was inoculated into 20 ml of defined medium containing either 10 mM or 10 µM MgCl_2_ and CaCl_2_. Following an 8 h incubation (with shaking), the cells were harvested and washed, and 1 ml of cell suspension was inoculated into a 50 ml tube containing 9 ml of either 0.85% (w/v) NaCl alone, or 0.85% (w/v) NaCl supplemented with polymyxin B or cecropin A at a final concentration of 50 µg/ml. The cells were incubated at 25°C for 10 minutes, diluted in 0.85% (w/v) NaCl and approximately 1,000 colony forming units (CFU) were plated on MM blood agar plates. Plates were incubated for 5 days at 25°C under microaerophilic conditions and the resulting colonies were counted. All assays were carried out in triplicate.

### Acid Resistance Assay

Wild type and *phoP* mutant strains of *S. glossinidius* were grown to mid-log phase in MM liquid medium. The cells were harvested by centrifugation at 5,000 × *g* for 10 minutes at 4°C and washed twice with an equal volume of 0.85% (w/v) NaCl. Approximately 2×10^8^ CFU were inoculated in 10 ml of MM medium at various pH levels (7.0, 5.0, 4.5 and 4.0). Following 1 h incubation, the cells were diluted in 0.85% (w/v) NaCl solution and approximately 1,000 CFU were plated on MM blood agar plates. Plates were incubated for 5 days at 25°C under microaerophilic conditions and the resulting colonies were counted. All assays were carried out in triplicate.

### Growth Curves

Wild type and *phoP* mutant strains of *S. glossinidius* were grown to mid-log phase in MM liquid medium. 5 ml of each culture was harvested by centrifugation at 5,000 × g for 10 minutes at 4°C. The cells were then washed twice with an equal volume of defined medium containing 10 µM MgCl_2_ and inoculated into 10 ml aliquots of defined medium containing either 10 mM or 10 µM MgCl_2_ and CaCl_2_. The cultures were maintained at 25°C, and measurements of turbidity (OD_600nm_) were obtained at 24 h intervals.

### *Salmonella enterica* Complementation Experiments

*Salmonella enterica* serovar Typhimurium EG9461 [27] and EG5931 [39] harboring pUHE21-2 lacI^q^ [55] or pUHE21-2 lacI^q^ derivatives were grown overnight in N-minimal medium containing 10 mM MgCl_2_. Overnight cultures were diluted 1∶100 in fresh N-minimal medium containing 10 mM MgCl_2_ and allowed to grow for 4 h. The cells were harvested by centrifugation at 8,000 × *g* for 3 min at 4°C, washed twice with 0.85% (w/v) NaCl, and resuspended in N-minimal medium containing either 10 mM or 10 µM MgCl_2_. Following 3 h of growth, β-galactosidase activity was determined as described by Miller [56].

### Total Lipid Extraction and Thin Layer Chromatography

Wild type and *phoP* mutant strains of *S. glossinidius* were grown to mid-log phase in MM liquid medium. 200 ml of each culture was harvested by centrifugation at 5,000 × *g* for 10 minutes at 4°C. The cells were then washed twice with an equal volume of 0.85% (w/v) NaCl. After the second wash, the cells were harvested as described above and resuspended in 5 ml of 0.85% (w/v) NaCl. 2.5 ml of each cell suspension was inoculated into 200 ml of defined media containing either 10 mM or 10 µM MgCl_2_ and CaCl_2_, and the cultures were incubated for 8 h with shaking. Following incubation, the cells were harvested by centrifugation at 5,000 × *g* for 10 minutes at 4°C and washed with an equal volume of 0.1% (w/v) ammonium acetate. The cell suspensions were centrifuged as described above and resuspended at a final concentration of 3×10^11^ CFU/ml. 200 µl of each cell suspension was used for total lipid extraction in accordance to the Folch method [57]. Lipid extracts were spotted on a 20×20 cm C_18_ thin layer chromatography plate (Whatman) and developed twice with chloroform: methanol: water (60∶30∶5, by volume). After chromatography, the plate was allowed to dry and lipids were visualized by iodine staining.

### RNA Isolation and Quantitative PCR Analyses

RNA was prepared using the SV Total RNA Isolation System (Promega). Following RNA purification, sample aliquots were treated with Turbo DNase (Ambion) to remove contaminating DNA. RNA samples were then reverse transcribed using the Maxima First Strand cDNA Sysnthesis kit (Fermentas). Quantitation of cDNA was performed in triplicate using the Maxima SYBR Green/ROX qPCR Master Mix (Fermentas). Samples were analyzed using an iCycler iQ Multicolor Real-Time PCR Detection System (Bio-Rad). Relative transcript levels were estimated using the standard curve method, with expression levels normalized against a gene encoding a ribosomal protein (*RplB*) that is expressed constitutively in *S. glossinidius* [8]. A list of the primer sets and respective target genes is presented in Table S1.

### Microinjection of *Sodalis glossinidius* into Louse and Tsetse Flies

*Glossina morsitans morsitans* Westwood tsetse flies were maintained at the Institute of Tropical Medicine (Antwerp, Belgium) as described previously [25]. *Pseudolynchia canariensis* hippoboscid louse flies were maintained on a pigeon colony at the University of Utah. Mid-log phase *S. glossinidius* cells were collected in a 1.5 ml tube by centrifugation at 8,000 *g* for 2 min and resuspended in PBS. Insects were injected into the ventral thorax with ≈ 2×10^4^ CFU of the *S. glossinidius phoP* mutant strain, *fliM* mutant strain [24], serving as a positive control for tsetse flies, or *fliM* and wild type strain (serving as a positive controls for louse flies). Microinjections were also performed with heat-killed wild type and *fliM* mutant strains (80°C for 15 min) to serve as negative controls. Insects were sacrificed for DNA isolation at various times post-injection and the resulting DNA samples were screened for the presence of bacteria using PCR primers specific for *S. glossinidius* wild type and the chloramphenicol resistance markers of the mutant strains.

## Supporting Information

Figure S1 A. Sequence alignment of PhoQ homologues derived from *S. glossinidius* and related Gammaproteobacteria. The box highlights the PhoQ acidic cluster (acidic residues are shaded in red) that is known to be involved in magnesium binding [17], [38], [47]. The PhoQ homologues of the insect endosymbionts *S. glossinidius* and *A. nasoniae* and the PhoQ homologue of the soft-rot plant pathogen *D. dadantii* have accumulated non-acidic amino acid substitutions within this cluster, suggesting that these proteins have a reduced ability to bind to magnesium and mediate the repression of PhoP-activated genes. Notably, the S. *glossinidius* PhoQ homologue also has a hydrophobic phenylalanine (shaded in blue) at a charged position (histidine 120) that is required for magnesium binding and magnesium-mediated repression in the PhoQ of *S. enterica* [38]. The alignment was generated using the online MAFFT tool [59]. B. Ribbon representation of the monomeric crystal structure of *S. typhimurium* PhoQ. Red-colored side chains represent acidic residues and gray spheres represent magnesium ions. Missing acidic residues in the *S. glossinidius* PhoQ sequence (D149 to D151) are highlighted. The structural diagram was generated using Pymol software (http://www.pymol.org/).
(TIF)

Figure S2 Schematic illustrating construction of replacement alleles for *S. glossinidius* recombineering. The drug resistance marker (chloramphenicol acetyltransferase, *cat*) was amplified using PCR primers with 5′-flanking sequences that match 35 base target sequences in the *S. glossinidius* chromosome. This PCR product was then integrated into a plasmid borne copy of the target gene, via lambda-Red mediated homologous recombination [52]. After selection for integration, the plasmid was used as PCR template for the synthesis of the replacement construct.
(TIF)

Table S1 Sequences of oligonucleotide primers used in quantitative PCR experiments.
(DOC)
