# Supplementary figures and images for: Attenuation of the Sensing Capabilities of PhoQ in Transition to Obligate Insect–Bacterial Association
Source: PLoS Genet. 2011 Nov 3;7(11):e1002349. doi: 10.1371/journal.pgen.1002349 (PMC3207850; doi:10.1371/journal.pgen.1002349)

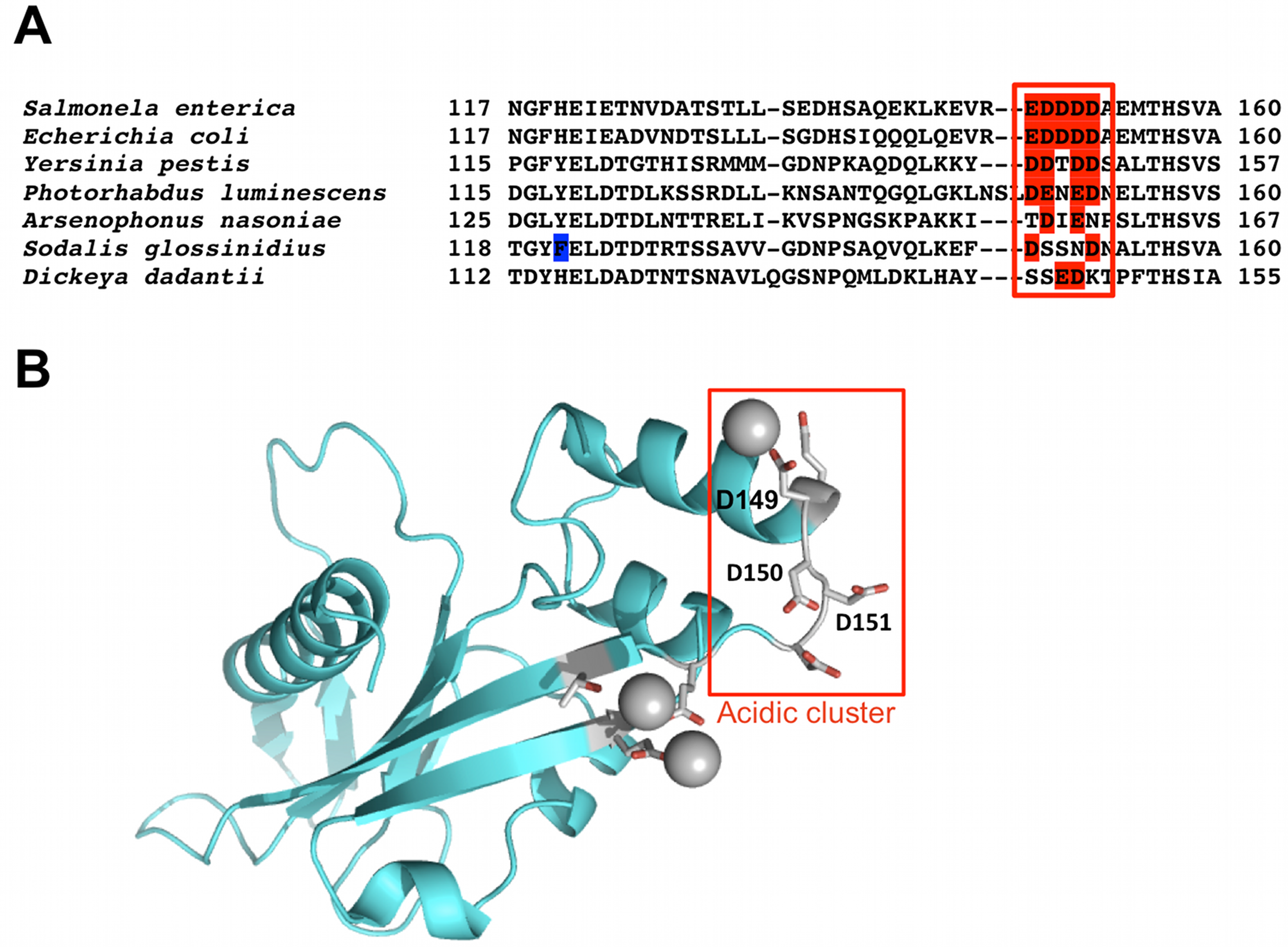

Supplement: Figure S1 — A. Sequence alignment of PhoQ homologues derived from S. glossinidius and related Gammaproteobacteria. The box highlights the PhoQ acidic cluster (acidic residues are shaded in red) that is known to be involved in magnesium binding [17], [38], [47]. The PhoQ homologues of the insect endosymbionts S. glossinidius and A. nasoniae and the PhoQ homologue of the soft-rot plant pathogen D. dadantii have accumulated non-acidic amino acid substitutions within this cluster, suggesting that these proteins have a reduced ability to bind to magnesium and mediate the repression of PhoP-activated genes. Notably, the S. glossinidius PhoQ homologue also has a hydrophobic phenylalanine (shaded in blue) at a charged position (histidine 120) that is required for magnesium binding and magnesium-mediated repression in the PhoQ of S. enterica [38]. The alignment was generated using the online MAFFT tool [59]. B. Ribbon representation of the monomeric crystal structure of S. typhimurium PhoQ. Red-colored side chains represent acidic residues and gray spheres represent magnesium ions. Missing acidic residues in the S. glossinidius PhoQ sequence (D149 to D151) are highlighted. The structural diagram was generated using Pymol software (http://www.pymol.org/). (TIF) [file pgen.1002349.s001.tif]

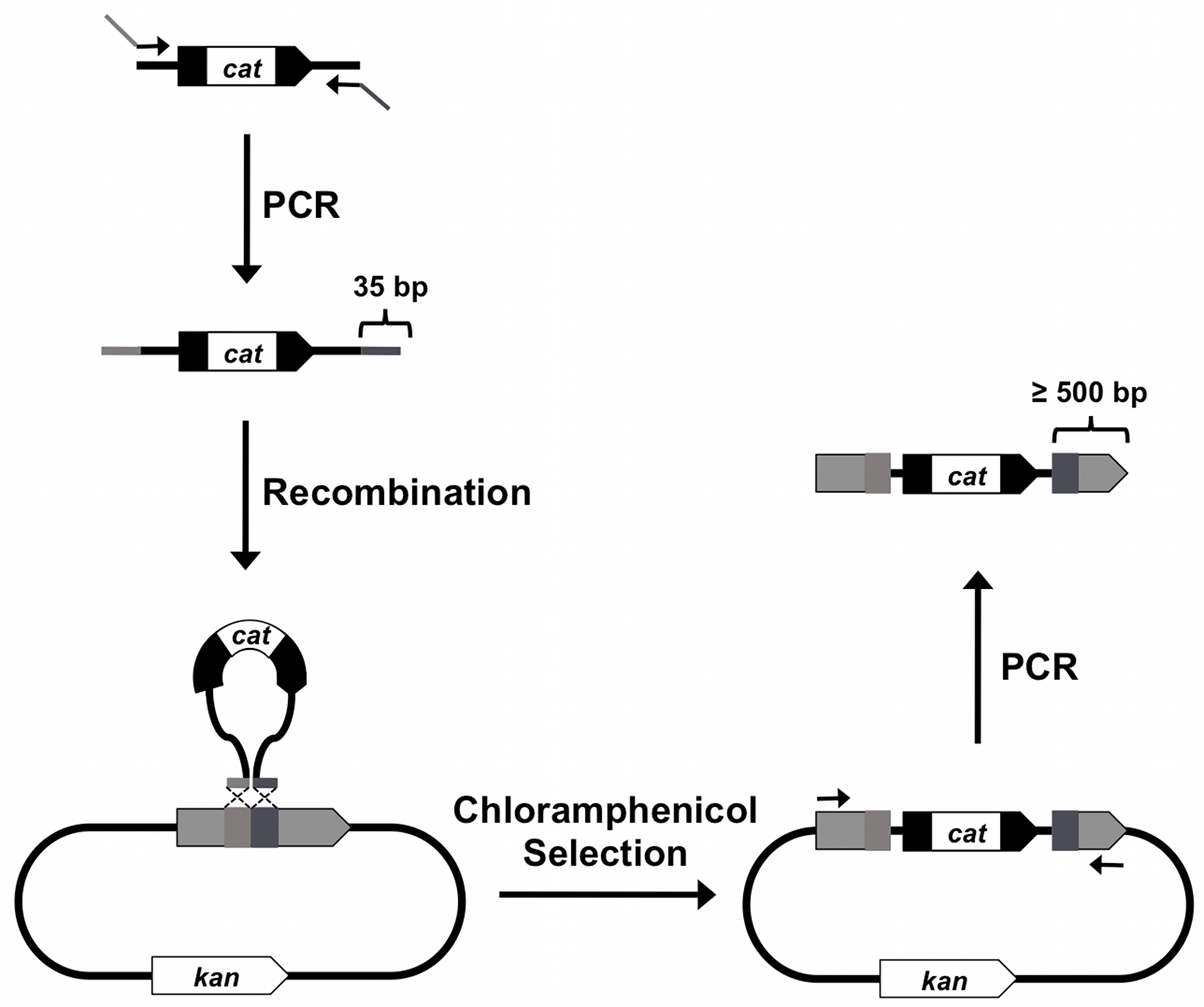

Supplement: Figure S2 — Schematic illustrating construction of replacement alleles for S. glossinidius recombineering. The drug resistance marker (chloramphenicol acetyltransferase, cat) was amplified using PCR primers with 5′-flanking sequences that match 35 base target sequences in the S. glossinidius chromosome. This PCR product was then integrated into a plasmid borne copy of the target gene, via lambda-Red mediated homologous recombination [52]. After selection for integration, the plasmid was used as PCR template for the synthesis of the replacement construct. (TIF) [file pgen.1002349.s002.tif]
