# Supplementary material for: Attenuation of the Sensing Capabilities of PhoQ in Transition to Obligate Insect–Bacterial Association
Source: PLoS Genet. 2011 Nov 3;7(11):e1002349. doi: 10.1371/journal.pgen.1002349 (PMC3207850; doi:10.1371/journal.pgen.1002349)
Supplement: Table S1 — Sequences of oligonucleotide primers used in quantitative PCR experiments. (DOC) [file pgen.1002349.s003.doc]

**Table S1**.

| **Target gene** | **Primer name** | **Sequence (5’ 🡪 3’)** |
| --- | --- | --- |
| *rplB* | QrplB F1 | TTCAGATCGTGGCTCGTGATGG |
|  | QrplB R1 | GCAGCATGTGTTCGGCATTACC |
| *pagP* | QpagP F1 | TCATTGTAACGGCGGATATGC |
|  | QpagP R1 | GCTGTGGCAACGCTTTACG |
| *pmrH* | QpmrH F1 | GCAGGTCATATTCTTGATGG |
|  | QpmrH R1 | CGAACGCTACGGTATTCC |
| *pmrE* | QpmrE F1 | TAATAGCAGCCTGACCTTCG |
|  | Qpmre R1 | AAGCAAGTCCACCACATCC |
| *ysaE* | QysaE F1 | AACTACCTGCTATCGCTCATTG |
|  | QysaE R1 | CGCACGCCAGATTCTCATC |
| *ysaF* | QysaF F1 | GCGAGCCAATGAAGAATG |
|  | QysaE R1 | AATAACGAACACCGATTACG |
| *ysaH* | QysaH F1 | TACTGCTACTGTTGGGCTGTG |
|  | QysaH R1 | CCGTGTGCGATTACTGTTATGG |
| *sycB* | QsycB F1 | ACTACCGTCCTATGTTATATGC |
|  | QsycB R1 | CTCCAACTCCGCTAATGC |
| *hilA* | QhilA F1 | TGCTCGCCGCTGGGGTTG |
|  | QhilA R1 | CACCACCAAGATTCAGTTCGTAAC |
| *invF* | QinvF F1 | GCCGATATAGAGCAGTGGATG |
|  | QinvF R1 | TGAGAATAGGACAAGCCGTAAC |
| *prgH* | QprgH F1 | GCGGTGAAGGTGGTGAGC |
|  | QprgH R1 | CGATGTGGATGCGATGAAGC |
| *sicA* | QsicA F1 | ATGGAAGGTGGTAAATAGC |
|  | QsicA R1 | ATCGTCATTATCGTCAGC |
| *sigE* | QsigE F1 | GCCTGTATGAAGCACTCGGTCTC |
|  | QsigE R1 | GCCGCTCGCTGTTGTCTGG |
| *ssaB* | QssaB F1 | GCAAAGGCTGGCGACAAC |
|  | QssaB R1 | CACATCCTGACATCCATGATTATG |
| *ssaH* | QssaH F1 | CCTTGCCCTGTCTGATTCC |
|  | QssaH R1 | AATAGCCTTCAGCGTCTCG |
| *ssaR* | QssaR F1 | TACGCTATTTCCACCAACTG |
|  | QssaR R1 | CATCGCCAATAACACATTCG |
| *ssaV* | QssaV F1 | CGTAACCAACTGGCTAAC |
|  | QssaV R1 | GCAATATCACCGAGAAGG |
| *sseB* | QsseB F1 | GGTGGACCCGACATTGCC |
|  | QsseB R1 | GCCATATCCTTAGTGCTTCTTGC |
| *ssrB* | QssrB F1 | TCTTCGGTATGGGCGGTATAG |
|  | QssrB R1 | GCACATTGATGACGGTCTGG |
